# Effects of atipamezole and medetomidine administration on seminal variables and functions of erection and ejaculation of the collared peccary (*Tayassu tajacu*) after electroejaculation

**DOI:** 10.1186/s12917-014-0170-2

**Published:** 2014-08-08

**Authors:** Ariana LC de Paiva, Talyta L Nunes, Maria GC de Oliveira, Alessandro ML de Morais, Érika AA dos Santos, Alexandre R Silva, Moacir F de Oliveira, Valéria V de Paula

**Affiliations:** 1 Laboratory of Anesthesiology and Animal Surgery, Universidade Federal Rural do Semi-Árido, Mossoró, RN, Brazil; 2 Laboratory of Animal Germplasm Conservation, Universidade Federal Rural do Semi-Árido, Mossoró, RN, Brazil; 3 Center multiplication of wild animals, Universidade Federal Rural do Semi-Árido, Mossoró, RN, Brazil

**Keywords:** Alpha-2 agonist, Alpha-2 antagonist, Propofol, Semen, Javelina

## Abstract

**Background:**

Alpha adrenergic drugs are usually used in the treatment of erectile and ejaculatory dysfunction in humans. The influence of such drugs on the seminal characteristics of wild animals has not been verified; whereas their impact on the seminal characteristics and erectile and ejaculatory functions of collared peccaries (*Tayassu tajacu*) has already been determined. This study aimed at investigating and comparing the effects of medetomidine and atipamezole on the seminal variables of collared peccaries undergoing electroejaculation as well as at determining whether these drugs affected the erectile and ejaculatory functions of this species.

**Results:**

A statistically significant difference in sperm concentration was observed between AP (100.0 ± 26.0 × 10^6^ sperm/ml) and MP (220.2 ± 49.8 × 10^6^ sperm/ml); however, both these treatments did not differ from P treatment (180.0 ± 50.7 × 10^6^ sperm/ml). No statistically significant difference was observed among all treatments with regard to erectile function. With regard to ejaculation time, no significant difference was observed between the MP and AP treatments; however, when compared with the P treatment, AP exhibited a significantly higher difference.

**Conclusions:**

When collared peccaries were anesthetized with propofol, neither medetomidine nor atipamezole significantly affected the characteristics of the semen or the erectile function, despite the fact that the AP treatment increased ejaculation time. Therefore, the data indicate that using propofol alone is an effective anesthetic protocol for collecting semen in collared peccaries. Other non-injectable anesthetic drugs, such as inhaled anesthetics, may be used in future research to collect semen from peccaries.

## Background

In order to ensure the successful application of reproductive biotechnologies to certain species, it is essential to obtain data on their reproductive physiology [1]. In this context, electroejaculation has been used for the collection of semen in wildlife [2],[3], especially in the collared peccary (*Tayassu tajacu*), which is an endangered species in the Caatinga and Atlantic Forest biomes [4]. Important results are obtained, especially when semen collection by electroejaculation is significantly more successful with appropriate anesthetic protocols [5]. Thus, anesthesia is necessary, not only to immobilize animals but also for their welfare [6],[7].

Propofol is considered a safe anesthetic for both the induction and maintenance of anesthesia in collared peccaries [8],[9], and it can also serve as a clinical treatment for erectile dysfunction [10]. Pre-anesthetic drugs, such as medetomidine and atipamezole, are used in anesthetic protocols in different domestic and wild species due to their excellent sedative effects. They have been used to preanesthestize raccoons [11], camels [12], reindeers [13], and cats [14]. However, the implications of their application to the reproductive tract have not been investigated in wild species.

Several anesthetic agents interfere with the mechanisms that control erectile function, thus emphasizing the need for a careful selection of anesthetics. Opioid analgesics; anticholinergics; anticonvulsants; antihypertensives such as alpha and beta blockers; and tranquilizers such as phenothiazines, butyrophenones, and benzodiazepines may contribute to or even cause erectile dysfunction [15]. Many drugs affect not only the erection but also the final sperm concentration, as physiological events that are related to the phase of seminal emission are significantly affected by the antagonism of alpha-1 receptors or the blockage of calcium channels, reducing the contractility of the vas deferens and seminal vesicle [16].

Since 1989, studies have been conducted on the collared peccary, and their spermatogenesis has been described [17]. Subsequently, the seminal evaluation of this species was performed with tiletamine/zolazepam administration as the anesthetic protocol [18], and later, propofol was compared with a treatment of acepromazine/tiletamine/zolazepam [19]. Most recently, protocols including acepromazine/ketamine [20] and propofol alone [3] have been used for the collection of semen in this species.

In a few studies conducted on electroejaculation in collared peccaries, the semen was tested after electroejaculation to see whether the pre-anesthetic or anesthetic drugs used for sedation, restraint, or anesthesia correlated with differences in sperm quality [3],[5],[18],[20]. The aim of this study was to compare the effects of medetomidine and atipamezole administration on the seminal variables of collared peccaries undergoing electroejaculation estimulation and to determine whether these drugs affected the erectile or ejaculatory function in this species.

## Methods

Experimental protocols and animal care were approved by Ethics Committee on Animal Use of UFERSA and were registered at the Chico Mendes Institute for Biodiversity Conservation (ICMBio-IBAMA) (no. 32480–1).

### Animal preparation

Twelve sexually mature male collared peccaries aged 21 (±1) months each and weighing 20 (±0.1 kg) were used. The animals belonged to the Center of Multiplication of Wild Animals at the Universidade Federal Rural do Semi-Árido, which is located in semiarid northeast Brazil (Mossoro, Rio Grande do Norte, Brazil).

The males were isolated from the females at three months before the commencement of the study and were maintained under a natural photoperiod (12 hours/day). They were kept outdoors in paddocks in groups of three animals and were fed a diet consisting of corn (79.8%), soybeans (15.4%), wheat bran (1.45%), calcium (2.6%), vitamins (0.2%), and other minerals (0.05%) complemented by tropical fruits. Water was freely available. Commencing at 20 hours before the experiments, animals were not allowed to drink water, and at 12 hours before the experiment, they were also put off feed.

On the day of the experiment, the animals were captured and restrained by netting. Then, the cephalic vein was catheterized (22-gauge catheter) and isotonic saline solution was administered at a rate of 5 ml/kg/min until the end of the electroejaculation procedure. Each animal underwent all three treatments, one at a time, being used as the control for themselves after a 15-day (washout) interval. Twelve repetitions were made for each treatment.

### Treatments

The sequence of administration of treatments was random, but twelve repetitions for each treatment were performed. The control treatment, or P treatment, was the one in which the animals received only anesthesia with propofol (Propovan®, Cristália, São Paulo, Brazil) at a dose of 5 mg/kg IV and no pre-anesthesia drug. The second treatment, the MP treatment, consisted of medetomidine (Medetor®, Virbac, Burgdorf, Germany) at a dose of 40 μg/kg IV and, subsequently, propofol (Propovan®, Cristália, São Paulo, Brazil) at a dose of 5 mg/kg IV. The third treatment, the AP treatment, was a dose of 200 μg/kg IV of atipamezole (Revertor®, Virbac, Burgdorf, Germany) followed by propofol (Propovan®, Cristália, São Paulo, Brazil) at a dose of 5 mg/kg IV.

### Maintenance of the anesthesia

In the experiments with all protocols, when the animal showed signs of awakening, one-fourth of the induction dosage of propofol was administered in bolus to keep the animal in a superficial anesthetic state as previously reported [19].

### Semen collection

The semen samples were collected using an electroejaculator (Autojac®, Neovet, Campinas, São Paulo, Brazil) that was connected to a 12-V source. The stimulatory cycle comprised 10 stimuli at each voltage, from 5 V up to 12 V and in increments of 1 V. Each electrical stimulus lasted for 3 s with breaks of 2 s after each stimulus. The stimuli were maintained for a period of 10 min. The electroejaculator probe containing linear electrodes measured 15 cm in length and 1.3 cm in diameter; 12 cm of the probe were inserted into the rectum of each peccary [19]. The semen was collected in plastic tubes and evaluated immediately.

### Erection evaluation

The erection was evaluated concurrently with the onset of electroejaculation. The presence or absence of an erection and whether the exposure of the penis was complete or not were recorded.

### Evaluation of the time of ejaculation latency

The ejaculation time was measured from the commencement of electroejaculation till the start of ejaculation.

### Semen evaluation

The semen volume was measured using micropipettes. The sperm motility (%) and vitality (0–5) were immediately assessed using light microscopy under 100x and 400x magnification. The percentage of live spermatozoa was determined by analyzing a slide that was stained with Brome Phenol Blue under light microscopy (400x); 200 cells per slide were counted. After the initial assessment, a 10 μL semen aliquot was diluted in 10% buffered formalin (2 mL) in order to kill and imobilize the spermatozoa, and the sperm concentration was determined using a Neubauer counting chamber. The functional integrity of the sperm membrane was evaluated by a hypoosmotic swelling (HOS) test in which distilled water (0 mOsm/L) was used as the hypoosmotic solution, and a 0.01 mL semen aliquot was diluted in a 0.09 mL hypoosmotic solution and kept in a water bath at 38°C. After 45 min, an aliquot of semen was placed on a glass slide, covered by a coverslip, and evaluated by microscopy (400×), counting 200 cells. Spermatozoa presenting swollen coiled tails were considered as representing a functional sperm membrane [19].

### Cystocentesis

At the end of electroejaculation, the animal was placed in dorsal recumbancy on the table for ventral hair removal and sterile skin preparation of the abdomen for urine sampling. After localizationof the bladder by means of ultrasonography (Aquila Vet, Pie Medical®, Nutricell, São Paulo, Brazil) with an 8 MHz-frequency microconvex transducer, real-time B-mode, a 16-gauge catheter was introduced into the bladder, aspirating the urine with the aid of a disposable 20 mL syringe. The urine sample was collected immediately and analyzed by evaluating the presence or absence of sperm cells and the concentration thereof. The cystocentesis was done to verify whether any treatment could promote sperm flow into the urinary bladder of the peccary after electroejaculation.

### Statistical analyses

The analyis was performed using SAS statistical software (SAS Institute Inc., Cary, North Carolina, USA) version 9.0 for Windows and SigmaPlot (SigmaPlot, Systat Software Inc.) version 12.0, and data were expressed as the mean ± the standard error. All data were primarily assessed for normality using the Shapiro–Wilk test, and the homogeneity of variance was assessed by the Bartlett test. Whenever necessary, logarithmic transformation was used for determining the parametric assumptions. An arccosine transformation was used in the case of variables expressed in percentages; that is, motility, functional integrity of the sperm membrane, and live spermatozoa. However, in the tables, the results were presented without statistical transformation. The semen parameters were analyzed using a one-way ANOVA for repeated measures, and this was followed by the Tukey test. The evaluation of erection and the time of ejaculation latency were analyzed by Cochran’s Q test and McNemar’s test in accordance with the architecture of the data. For all the statistical analyses, significance was defined as P < 0.05.

## Results

When the treatments were evaluated for their ability to promote erection, no statistically significant difference was present among any of the treatments (Table 1).

**Table 1:** **Mean values ± SE for the percentage of collared peccaries (*Tayassu tajacu*) that had an erection according to different anesthesia protocols**

	**P**		**AP**		**MP**	
	**n**	**%**	**N**	**%**	**n**	**%**
Erection						
Yes	9	75	8	66.7	7	58.3
No	3	25	4	33.3	5	41.7

There was no significant difference in the results between treatments.

P-propofol alone; MP-medetomidine/propofol; AP-atipamezole/propofol treatments.

With regard to ejaculation time, no significant difference was observed between the MP and AP treatments; however, when both these were compared with the P treatment, the AP treatment exhibited a significant difference: The time required for ejaculation with the AP treatment was significantly longer than that for the other treatment (Table 2).

**Table 2:** **Mean values ± SE for the ejaculation latency time (minutes) of the collared peccaries (*Tayassu tajacu*) that underwent electroejaculation according to different anesthesia protocols**

	**Mean ± SE**	**Minimum**	**Maximun**
P	2.66 ± 1.41^BC^	1.0	5.0
AP	4.27 ± 2.45^A^	1.0	9.0
MP	3.9 ± 2.42^AB^	1.0	8.0

^A-C^Values with different superscripts differ significantly in column (P < 0.05).

P-propofol alone; MP-medetomidine/propofol; AP-atipamezole/propofol treatments.

Variables such as ejaculate volume, sperm motility, vitality, and functional integrity of the membrane did not present significant differences between the three treatments (P > 0.05). Only the sperm concentration exhibited a significant difference between the three treatments (P > 0.05) (Table 3). The MP treatment group showed a sperm concentration that was significantly higher than that in the AP treatment group (P < 0.05), but neither group exhibited a statistically significant difference when compared with the P treatment group (P > 0.05).

**Table 3:** **Mean values ± SE of semen parameters of collared peccaries (*Tayassu tajacu*) according to different anesthesia protocols**

	**P**		**MP**		**AP**	
	**Mean ± SE**	**Range**	**Mean ± SE**	**Range**	**Mean ± SE**	**Range**
Volume (μL)	2520.0 ± 603.1^a^	0-7000.0	1640 ± 329.3^a^	0-3500.0	1454.9 ± 784.3^a^	50-9000.0
Motility (%)	90.5 ± 3.1^a^	70-100	84.5 ± 6.3^a^	30-95	84.6 ± 4.2^a^	60-100
Vigor	3.7 ± 0.5^a^	3-5	3.5 ± 0.5^a^	2-5	4.0 ± 0.3^a^	2-5
Alive (%)	65.10 ± 7.0^a^	29-98	72.8 ± 3.1^a^	58-88	71.27 ± 4.4^a^	0-95
Concentration( x 10^6^ sperm/mL)	180.0 ± 0.7^ab^	10-510	220.2 ± 49.7^a^	30-550	100.0 ± 25.86^b^	20-280
Functional integrity of the membrane (%)	82.3 ± 7.9^a^	20-95	80.50 ± 4.2^a^	57-98	88.5 ± 2.7^a^	69-95

^a-b^Values with different superscripts differ significantly in line (P < 0.05).

P-propofol alone; MP-medetomidine/propofol; AP-atipamezole/propofol treatments.

Sperm were present in the urine samples from all three treatments. The sperm concentrations in the urine samples differed between the MP treatment (13.0 ± 3.2 × 10^6^ sperm/mL of urine) and the AP treatment (2.3 ± 1.8 × 10^6^ sperm/mL of urine). However, neither of these treatments exhibited a statistically significant difference when compared with the P treatment (5.2 ± 3.6 × 10^6^ sperm/ml of urine).

## Discussion

To the best of the authors’ knowledge, this is the first study that correlates different preanesthetic drugs administered with the semen parameters of collared peccaries.

Sperm concentration values obtained in this study were higher than those reported in collared peccaries anesthetized with acepromazine/tiletamine/zolazepam (13.8 ± 5.7 × 10^6^ sperm/mL) [19]. The lower sperm concentration obtained by these researchers may be related to the use of acepromazine. Alpha-1 receptors are responsible for normal contractility of the vas deferens and the emission of semen [21]; hence, alpha receptor antagonist drugs (acepromazine, selective alpha-1 blocker, and atipamezole) can affect the final sperm concentration.

Sperm concentrations similar to those reported in this study were obtained (118.0 ± 158.4 × 106 sperm/mL) by using a propofol-alone protocol [19]. An increase in sperm concentration after the administration of the MP treatment was also observed when evaluating the semen of domestic cats that were pre-anesthestized with medetomidine [22].

The difference in sperm concentration between the AP treatment and the MP treatment can be explained by the interaction between the alpha-2 agonist or antagonist drug and the sympathetic nervous system. The sympathomimetic effect is mainly due to the effect of the agonist drug on the alpha-2 postsynaptic receptors as well as on the alpha-1 receptors, although these drugs have a high selectivity for alpha-2 receptors [23]. The activation of alpha adrenergic receptors promotes the contraction of the cauda epididymis, the emission of sperm, and, therefore, the increased sperm concentration [24]. The antagonist drug blocks the postsynaptic alpha-2 adrenergic receptors, thereby decreasing sympathetic (adrenergic) activity and increasing parasympathetic (cholinergic) activity. The ejaculation stimulus generator responds to the sympathetic spinal control, whereas the parasympathetic control induces the erection phase [25].

The penis receives neural input from two sympathetic sources. The hypogastric nerve arises from the caudal mesenteric ganglion (derived from the sympathetic chain at T12-L5) and joins the pelvic nerve to form the pelvic plexus, beyond which the cavernous nerve runs directly to the penis. Other sympathetic fibers run caudally in the sympathetic chain to S1-S3, where they exit with the pudendal and pelvic nerves [26].

In erectile tissue, both alpha-1 and alpha-2 and beta-2 adrenoceptors are expressed, and they probably mediate vasodilation in response to the increase in adrenaline during erection [27]. In essence, ejaculation occurs through the forceful contractions within accessory sex organs, including the vas deferens, seminal vesicle, and prostate. Is integrated via the sympathetic nervous system, and it has long been known that the alpha-1 adrenoceptor plays an important role in the regulation of the motility of accessory sex organs [28]. The blocking of alpha-1 adrenoceptors would lead to muscular paralysis of these organs and, hence, prevent sperm transport. The alpha-1 adrenoceptors, particularly alpha-1A adrenoceptors, are required for normal contractility in the vas deferens and consequent sperm ejaculation and, hence, have a function in fertility [20].

The data presented in this study on the concentration of sperm in the urine after electroejaculation are the first published for the collared peccary. The average concentration of sperm observed in the urine for the P treatment in this study is similar to the values found in pigs (3.16 ± 9.2 × 10^6^ sperm/ml of urine) pre-anesthetized with tiletamine/zolazepam after electroejaculation [29]. This retrograde flow of sperm into the bladder was also found in dogs subjected to electroejaculation after the administration of xylazine (similar to medetomidine, which is an alpha-2 agonist) [30]. Sperm were found in all dog urine samples after ejaculation, despite the fact that the pretreatment with sympathomimetic agents significantly decreased the retrograde flow [31]. In contrast, no significant differences in the percentage of the retrograde flow of sperm were observed after the administration of xylazine in pigs [32] or medetomidine in cats [22]. The same authors also assert that a small amount of sperm can be found in the urine of cats after electroejaculation even without the use of a chemical restraint or an ejaculation stimulus. Higher sperm concentration in the collard peccaries’ urine for the MP treatment as compared with AP treatment can be attributed to the fact that alpha adrenergic blocking agents cause the relaxation of smooth muscle tone in the bladder neck, and this may contribute to retrograde ejaculation [33]. The blockage of alpha-1Aadrenoceptors, located at the bladder neck, leads to the reflux of seminal fluid from the prostatic urethra into the bladder [34]. The adrenergic blockage results in decreased prostatic smooth muscle tone and a consequent reduction in pressure of the prostatic urethra [35].

The lack of significant differences between treatments with regard to their ability to affect erection can be explained by the fact that the specific agonist and antagonist alpha-2 adrenergic drugs have little or no role in alpha-1 adrenoceptors. The alpha-2 adrenoceptor expressed in cavernous tissue has little functional activity in the erection [36]. Therefore, alpha-1 receptors, which are abundant in the erectile tissue, are directly involved in this function through the relaxation of vascular smooth muscle post-junctional adrenoceptors, as alpha-1 receptors are blocked [37].

No influence on ejaculation latency time was observed either in this study or in others [38] when an alpha-2 antagonist (such as yohimbine) was administered in rats. However, a reduction in ejaculation latency time was measured after the administration of yohimbine in dogs subjected to manual stimulation [39]. Previous research has shown that the central action of alpha-2 antagonist drugs can facilitate both excitement and copulatory events that are associated with the sexual behavior of males, suggesting that alpha-2 adrenoceptors are abundant in the nucleus of the brain and the hypothalamus and are also involved in regulating sexual behavior [40]. Since the animals in this study were anesthetized, the majority of such effects were likely eliminated by the anesthesia, which consequently also eliminated any behavioral responses similar to those observed in studies using unanesthetized subjects.

## Conclusion

When collared peccaries were anesthetized with propofol, neither medetomidine nor atipamezole significantly affected the characteristics of the semen or the erectile function, despite the fact that the atipamezole treatment increased ejaculation time. Therefore, the data indicate that using propofol alone is an effective anesthetic protocol for collecting semen in collared peccaries. Other non-injectable anesthetic drugs, such as inhaled anesthetics, may be used in future research to collect semen from peccaries.

## Competing interests

None of the authors of this article has a financial or personal relationship with other people or organizations that could inappropriately influence or bias the content of this article.

## Authors’ contributions

VVP conceived of the study, and participated in its design and coordination. ARS and EAAS contributed toward the semen analysis. MFO contributed toward the capture and welfare of animals in captivity. All authors, ALCP, TLN, MGCL, and AMLM, contributed to the acquisition and interpretation of data, drafting and revision of this article. All authors have read and approved the final article.
